# Binder‐Free 3D Integrated Ni@Ni_3_Pt Air Electrode for Zn–Air Batteries

**DOI:** 10.1002/gch2.201900027

**Published:** 2019-06-27

**Authors:** Thien Viet Pham, Yang Li, Wen‐Bin Luo, Hai‐Peng Guo, Xuan‐Wen Gao, Jia‐Zhao Wang, Hua‐Kun Liu

**Affiliations:** ^1^ Institute for Superconducting and Electronic Materials University of Wollongong Wollongong NSW 2522 Australia

**Keywords:** binder‐free, electrocatalysts, Ni_3_Pt alloy, pulsed laser deposition, rechargeable zinc–air batteries

## Abstract

Developing an air electrode with high efficiency and stable performance is essential to improve the energy conversion efficiency and lifetime of zinc–air battery. Herein, Ni_3_Pt alloy is deposited on 3D nickel foam by a pulsed laser deposition method, working as a stable binder‐free air electrode for rechargeable zinc–air batteries. The polycrystalline Ni_3_Pt alloy possesses high oxygen‐conversion catalytic activity, which is highly desirable for the charge and discharge process in zinc–air battery. Meanwhile, this sample technique constructs an integrated and stable electrode structure, which not only has a 3D architecture of high conductivity and porosity but also produces a uniform Ni_3_Pt strongly adhering to the substrate, favoring rapid gas and electrolyte diffusion throughout the whole energy conversion process. Employed as an air electrode in zinc–air batteries, it exhibits a small charge and discharge gap of below 0.62 V at 10 mA cm^−2^, with long cycle life of 478 cycles under 10 min per cycle. Furthermore, benefitting from the structural advantages, a flexible device exhibits similar electrochemical performance even under the bending state. The high performance resulting from this type of integrated electrode in this work paves the way of a promising technique to fabricate air electrodes for zinc–air batteries.

Efficient harvesting, storage, and utilization of renewable energy are the core of clean energy high efficient utilization research. Among various energy storage technologies, metal–air batteries possess unlimited potential and economic value due to their high theoretical energy densities and abundant resource in the earth.^1^, ^2^, ^3^ Particularly, zinc‐based batteries are considered as one of the most promising candidates for next‐generation energy storage system because of their nonflammable, environmentally friendly, and high economic efficiency.^4^, ^5^ During long‐term global research work, the challenge to realize long lifetime zinc–air battery (ZAB) stays in the exploration of appropriate air electrode for oxygen conversion reaction combined with satisfied catalytic activity and ideal electrode structure.^2^, ^6^, ^7^, ^8^, ^9^, ^10^, ^11^, ^12^, ^13^, ^14^ For example, issues of conventional particulate electrocatalyst detachment from the substrate and original designed air electrode destruction during long‐term cycling period seriously and directly affect the cycle‐life of the zinc–air battery. To address these issues, designing a stable and integrated air electrode is considered as one of the most feasible and efficient solutions. Meanwhile, electrocatalytic activity is the primary guarantee of possessing high energy density.^15^, ^16^ Platinum (Pt) alloyed with a less noble late transition 3d metal such as Fe,^17^, ^18^ Co,^19^ Ni,^20^, ^21^, ^22^, ^23^ and Cu,^24^, ^25^, ^26^ has been extensively studied and regarded as promising electrocatalysts for oxygen conversion due to the ligand effects and strain effects.^27^ It has been found that the Pt‐based alloy could exhibit enhancements in oxygen conversion reaction activity over Pt due to its weaker bonds to HO* than that of pure Pt.^6^, ^26^, ^28^, ^29^ Among the Pt‐based alloy research, effectively reducing the use of Pt and exploring a feasible technology are important research points for future high economic application.^30^, ^31^, ^32^, ^33^

In order to design an appropriate air electrode combined with stable electrode structure and high efficient catalytic activity, in this work, a polycrystalline Ni_3_Pt alloy thin film was uniformly deposited on 3D nickel foam to fabricate binder‐free air electrode by pulsed laser deposition (PLD) method, which can not only simplify the preparation process, but also address some issues such as the increasing contact resistance and lower catalyst usage efficiency resulting from the addition of polymeric binder. In addition, the low cost 3D porous structured nickel foam served as the current collector that can provide a continuous conductive network and ensure the accessibility of the electrolyte into the surface of the Ni_3_Pt alloy surface, further enhancing the electrode reaction kinetics. Based on the optimized electronic structure of Ni_3_Pt alloy and the merit of structure design of electrode, the zinc–air battery fabricated by this air cathode (denoted as Ni@Ni_3_Pt) can perform at a low charge and discharge gap (<0.62 V) for 478 cycles (10 min each cycle). This low overpotential is also encouraging to improve round‐trip efficiency of air batteries. Moreover, owing to the unique electrode design, the Ni@Ni_3_Pt‐based air cathode exhibits good mechanical stability, a flexible device was also successfully constructed, and the operated voltage has no obvious change at the bending state.

The schematic diagram as shown in Figure S1 (Supporting Information) indicates the whole experiment process. The element stoichiometry 3:1 of target is based on the powder mixture ratio of nickel versus platinum powder. First, the nickel foam was cleaned thoroughly with weak acid solution and ethanol and dried overnight in vacuum oven at 80 °C. Then, the nickel foam was transferred to PLD chamber to prepare Ni@Ni_3_Pt. The PLD coating was expected to form a layer of Ni_3_Pt on nickel foam which created a core–shell structure of Ni_3_Pt around Ni core. Field emission scanning electron microscopy (FE‐SEM) and high resolution transmission electron microscopy were used to analyze the structure of Ni@Ni_3_Pt. As shown in **Figure** **1**a, the obtained 3D porous network of Ni@Ni_3_Pt shows the similar structure as that of pure nickel foam. Meanwhile, the deposited thin film indicates standard face‐centered cubic (fcc) crystal structure, with (111), (200), and (220) diffraction planes. The distribution of elements in the alloyed structure was characterized by high‐angle annular dark field (HAADF)‐scanning transmission electron microscopy (STEM) energy‐dispersive X‐ray spectroscopy (HAADF‐STEM‐EDX), as shown in Figure 1c–g and Figure S2 (Supporting Information). The elements Ni and Pt show a similar homogeneous distribution over the entire area and the entire phase is a single alloy phase, with elements ratio of ≈3:1. Meanwhile, the whole area indicates a single alloy phase.

**Figure 1:**
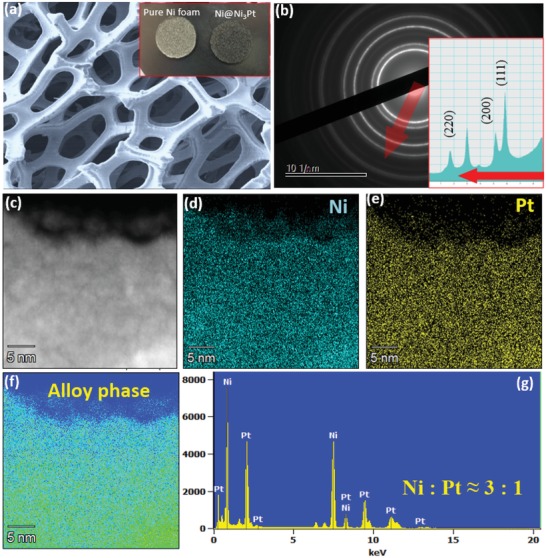
a) FESEM of the air electrode (photograph of nickel foam and Ni@Ni_3_Pt air electrode. b) Electron diffraction pattern of deposited Ni_3_Pt thin film. c–g) HAADF‐STEM energy‐dispersive X‐ray spectroscopy element mapping of deposited Ni_3_Pt thin film.

The deposited Ni_3_Pt thin film indicates an integrated continuous thin film structure with 15 nm thickness as shown in **Figure** **2**a,b. This polycrystal thin film structure is formed by a large amount of Ni_3_Pt nanocrystals in the range of 5 nm. The lattice fringes in Ni_3_Pt nanocrystals have measured spacings of 2.08 Å, which can be indexed to the (111) planes of face‐centered cubic. This planar spacing is smaller than that of fcc pure Pt, and they also provide evidence of the formation of the alloyed structure. Meanwhile, the defects in the crystal and strains among grain boundaries must be beneficial to the improvement of the catalytic activity. The chemical binding energy of the Ni 2p signal (observed at 867.5 and 853.0 eV) and Pt 4f signal (observed at 76.6 and 73.1 eV) in Figure 2e,f also indicates the coexistence of both metals.^34^, ^35^ The surface area of this electrode was measured by Brunauer–Emmett–Teller (BET). From the BET isotherm in Figure S3 (Supporting Information), the surface area is estimated to be 36.7 m^2^ g^−1^.

**Figure 2:**
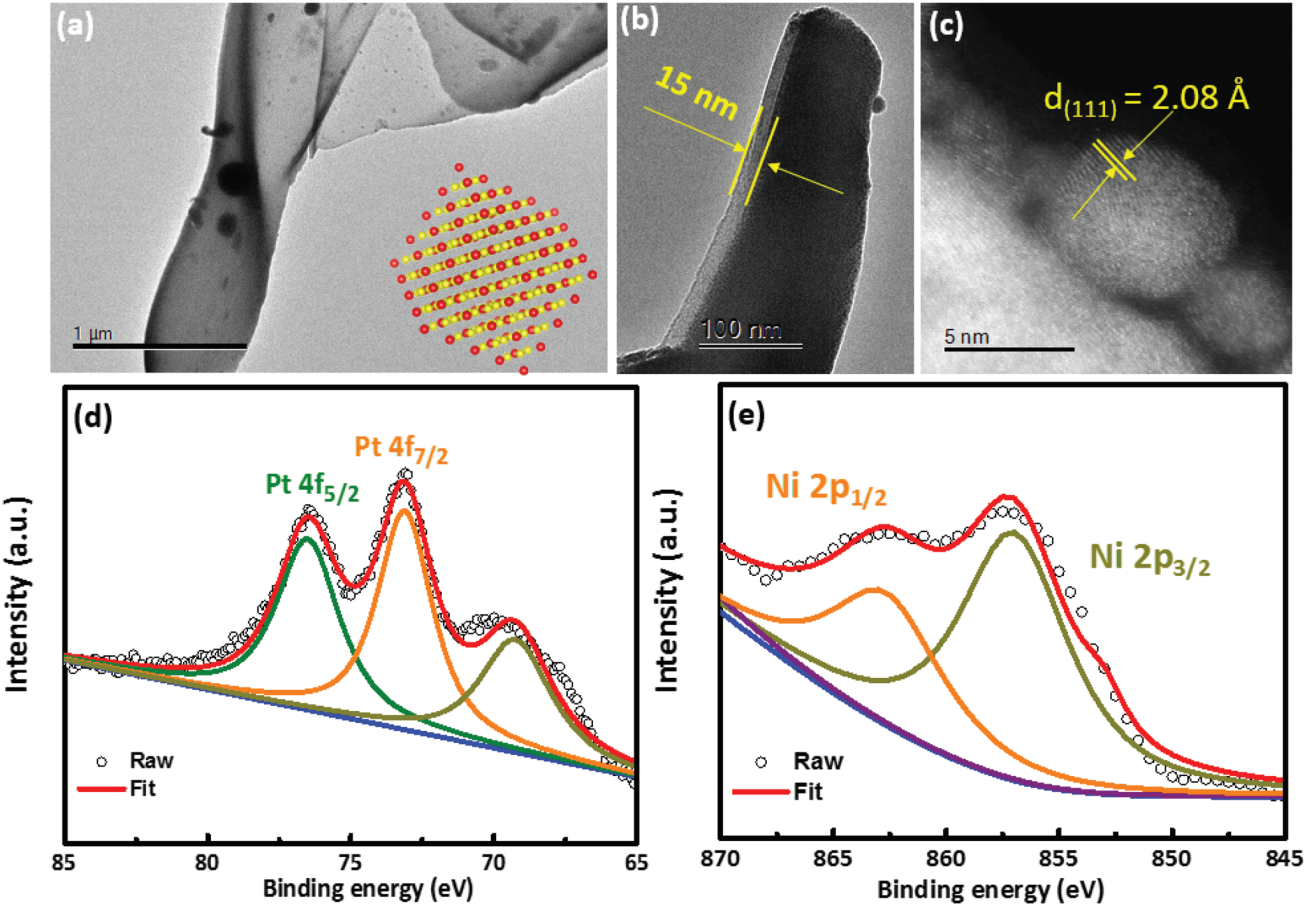
a,b) TEM of the deposited Ni_3_Pt thin film. c,d) HAADF‐STEM of deposited Ni_3_Pt nanocrystals. e,f) XPS spectra of Ni_3_Pt thin film.

The electrochemical performance of as‐prepared air electrode Ni@Ni_3_Pt was evaluated by zinc–air battery as shown in **Figure** **3**. Compared with commercial catalyst Ni@(Pt/C+IrO_2_), it is obviously concluded that reversible oxygen conversion reaction occurs in the whole reaction process as shown in Figure 3a. The charge and discharge polarization curves were recorded in Figure 3b. It clearly reveals that the Ni@Ni_3_Pt electrode not only has similar discharge curve with Ni@(Pt/C+IrO_2_), but also has lower charge potential than that of Ni@(Pt/C+IrO_2_) at any current density from 0 to 150 mA cm^−2^. This result indicates that the Ni@Ni_3_Pt can effectively accelerate the oxygen conversion reaction kinetics,^36^ The discharge and charge curves of Ni@Ni_3_Pt at the first cycle are given in Figure 3c. Ni@Ni_3_Pt shows a lower overpotential and slightly higher open circuit potential (1.31 V) than that of the state‐of‐art commercial Pt/C and IrO_2_ catalysts (1.29 V). The battery voltage and the power density are shown in Figure 3d. It can be seen that it can operate with the max power density as 90 mW cm^−2^ at the current density of 100 mA cm^−2^. In addition to the excellent charge and discharge capabilities, Ni@Ni_3_Pt shows good rechargeability as shown in Figure 3e, evidenced by 478 cycles over a duration of 79 h (6 cycles for 1 h), which ran at lower overpotential than that using Pt/C and IrO_2_ catalysts. To eliminate the influence of nickel foam (NF), the battery performance of ZAB using NF as the air cathode was recorded in Figure S4a,b (Supporting Information), which exhibits large voltage gap and low power density. These results are superior to those reported as shown in Table S1 (Supporting Information). Meanwhile, the discharge and charge voltage was stable for almost 80 h, and the discharge and charge voltage could be recovered with the electrolyte and zinc replaced.^37^, ^38^ After the replacement, battery lasted for another 154 cycles. The second replacement helped battery operate for 104 cycles. After the replacement of zinc, the battery discharge and charge voltage return to their original values (1.20 and 1.55 V), which indicate that the capacity is unchanged after zinc replacement.^7^ All of these results evident the superior stability of the air electrode. From the cycling curve, the overpotential always stays lower than 0.62 V, and the charge voltage is below 1.7 V. These overpotential and charge potential are much superior to those reported results summarized in Table S1 (Supporting Information). All of these strongly indicate a high bifunctional activity and excellent stability of Ni@Ni_3_Pt catalyst, which is essential for rechargeable zinc–air battery.

**Figure 3:**
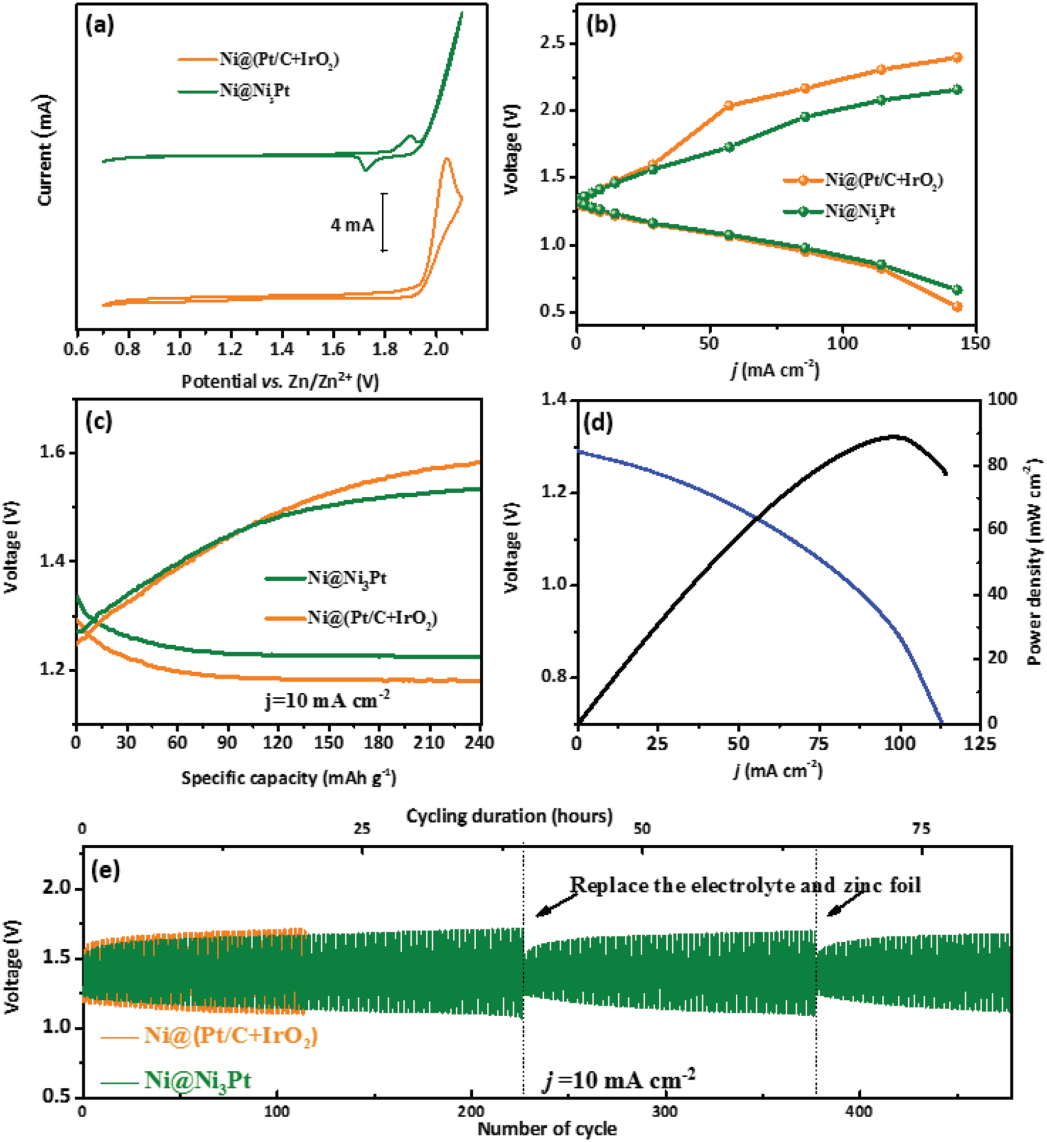
a) Cyclic voltammograms (CV) of Ni@Ni_3_Pt and Ni@(Pt/C+IrO_2_). b) Polarization and power density curves. c) Discharge–charge cycling curves at current density of 10 mA cm^−2^. d) Polarization and power density curves. e) Cycling performance at a charge–discharge current density of 10 mA cm^−2^ for Ni@(Pt/C+IrO_2_) and Ni@Ni_3_Pt electrodes, respectively.

A flexible device using Ni@Ni_3_Pt as air electrode was demonstrated in **Figure** **4** with flexible zinc foil as anode, carbon paper as gas diffusion layer, and alkaline gel electrolyte.^39^, ^40^ The alkaline poly(vinyl alcohol) (PVA) gel electrolyte is fabricated with KOH, Zn(Ac)_2_, and PVA in deionized water (see details in the Experimental Section), which is flexible and bendable, delivering considerable ionic conductivity and mechanical flexibility for zinc–air battery.^30^, ^40^ The two clips were used to hold all components of the flexible battery, as shown in Figure 4a,b. The bending condition at 57° is exhibited in Figure 4b. In Figure 4c, it exhibits the battery voltage as a function of current density under the normal and bending condition. The flexible device has the open potential of 1.375 V. At the current density of 1 mA cm^−2^, the bending battery can operate at 1.32 V and nonbending at 1.3 V. The higher operating voltage of the bending state can be attributed to the better compression of the whole battery under curving condition than in the normal state. These results reveal that the Ni@Ni_3_Pt air electrode has attractive potential in the rechargeable, flexible zinc–air battery and wearable devices. A compact layer of oxidized zinc species due to parasitic reaction on the zinc electrode surface was observed in Figure 4d,e with the formation of ZnO and Zn(OH)_2_. The phases of ZnO and Zn(OH)_2_ were confirmed by X‐ray powder diffraction (XRD) in Figure 4f. It is obvious that the anode zinc plate changes dramatically before and after cycling. Therefore, in addition to the optimization of cathode catalysts, strong efforts are also required to develop stable Zn‐based anodes in the future research for practical applications.^30^, ^39^, ^41^

**Figure 4:**
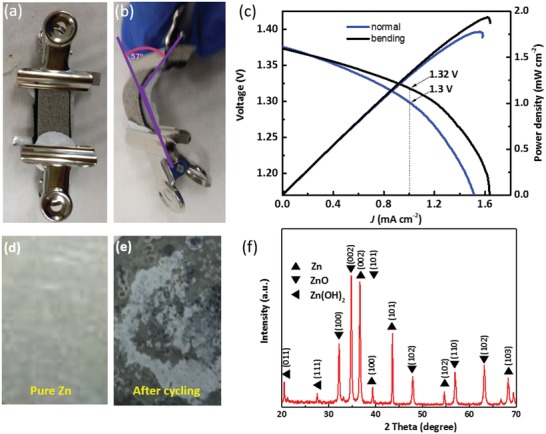
a,b) Photos and schematic diagram of the fabrication process of the flexible battery. c) Polarization and power density curves of the flexible battery. d,e) Zinc anode change before and after cycling. f) XRD pattern of the Zn plate after long‐term cycling.

In conclusion, polycrystalline Ni_3_Pt alloy thin film deposited on 3D microporous nickel foam (Ni@Ni_3_Pt) by PLD can be served as an integrated air electrode for zinc–air battery. It demonstrates excellent electrochemical performance including catalytic activity and cycling lifetime, delivering a maximum power density at 90 mW cm^−2^ and a stable discharge voltage (>1.09 V) at 10 mA cm^−2^. Owing to the stability of the integrated air electrode, it shows excellent cycling performance up to 478 cycles (>79 h) at a current density of 10 mA cm^−2^. Meanwhile, this integrated electrode is fabricated into a rechargeable flexible zinc–air battery. It shows impressive performance as well, with a high open‐circuit voltage of 1.375 V and a high operating voltage of 1.32 V at 1.0 mA cm^−2^ under bending state. Therefore, this work provides a promising technique to prepare appropriate air electrode in the future zinc–air battery research. The application of this integrated air electrode for zinc–air battery paves a new way for the development of high‐efficient, cost‐effective, and environmentally friendly energy conversion and storage devices.

## Experimental Section

*Synthesis of Ni_3_Pt on Ni Foam by PLD*: The targets were made by sintering a pressed mixture of platinum powder (<40 nm, Sigma) and nickel powder (−40 mesh, Sigma) according to the element stoichiometry at 700 °C for 6 h in argon gas. The laser outputs 248 nm wavelength, 30 ns pulses at a frequency that may be chosen between 1 and 10 Hz. The energy of this pulse was determined by choosing the pumping high voltage (HV) of the laser. The laser HV ranges from 24 to 32 kV and, depending on the state of the gas medium, will produce energies in the pulse between 400 and 1200 mJ. The rectangular output pulse of the laser has dimensions of ≈30 mm × 12 mm. The substrates for deposition included nickel foam to get air electrode, transmission electron microscopy (TEM) grids convenient for TEM operation.

*Characterization*: The corroded zinc anode was characterized by XRD (GBC MMA) with Cu Kα radiation that was operated over a 2θ range of 20°–70°. The morphological studies were performed by FE‐SEM (JEOL‐JSM‐7500). TEM energy‐dispersive X‐ray spectroscopy were used to investigate the Ni_3_Pt film on nickel foam.

*Electrochemical Measurements*: All the cells with coin cell (CR2032) type were assembled, using the Ni@Ni_3_Pt as cathode, a glass fiber separator, a polished zinc foil anode, and an aqueous electrolyte containing 6 m KOH + 0.2 m Zn(Ac)_2_. The CVs were obtained between 0.7 and 2.1 V at a scan rate of 0.2 mV s^−1^. In these tests, Ni@Ni_3_Pt and Ni@(Pt/C+IrO_2_) acted as the working electrodes, respectively. Ni@(Pt/C+IrO_2_) was prepared by drop casting 50 wt% Pt/C‐50 wt% IrO_2_ on nickel foam (the loading was 1 mg cm^−2^). On the other hand, zinc foil served as both counter and reference electrodes. All battery tests were carried out on LAND CT 2001A multichannel battery testers at room temperature in oxygen atmosphere. All the potentials throughout this paper were referred to the potential of the Zn/Zn^2+^ standard couple.

*Flexible ZAB Assembly*: A polished zinc foil (0.25 mm thickness) was used as anode. The air electrode was Ni@Ni_3_Pt mentioned above. The gel polymer electrolyte was prepared as follows: 1.0 g PVA powder (Sigma) was dissolved in 10.0 mL deionized water at 95 °C under magnetic stirring for 2.0 h. Then 1.0 mL of 18.0 m KOH filled with 0.20 m Zn(Ac)_2_ was added, and the electrolyte solution was kept stirring at 95 °C for 40 min. Then the solution was freezed, and then thawed at room temperature. The procedure was repeated twice to form gel electrolyte. Then the flexible ZAB was assembled with air electrode Ni@Ni_3_Pt and zinc foil placed on the two sides of PVA gel.^30^, ^37^

## Conflict of Interest

The authors declare no conflict of interest.

## Supporting information

Supplementary
