# Supplementary material for: Binder‐Free 3D Integrated Ni@Ni3Pt Air Electrode for Zn–Air Batteries
Source: Glob Chall. 2019 Jun 27;3(9):1900027. doi: 10.1002/gch2.201900027 (PMC6733491; doi:10.1002/gch2.201900027)
Supplement: Supplementary file 1 — Supplementary [file GCH2-3-1900027-s001.pdf]

Copyright WILEY-VCH Verlag GmbH & Co. KGaA, 69469 Weinheim, Germany, 2019.

Global Challenges
Open Access

Supporting Information

for *Global Challenges,* DOI: 10.1002/gch2.201900027

Binder-Free 3D Integrated Ni@Ni_3Pt Air Electrode for Zn–Air Batteries

Thien Viet Pham, Yang Li, Wen-Bin Luo, Hai-Peng Guo, Xuan-Wen Gao, Jia-Zhao Wang,* and Hua-Kun Liu*

Supporting Information

Binder-free 3D Integrated Ni@Ni_3Pt Air Electrode for Zn-Air Battery

Thien Viet Pham[‡], *Yang Li*[‡], *Wen-Bin Luo*[*], *Hai-Peng Guo, Xuan-Wen Gao, Jia-Zhao Wang*[*], *and Hua-Kun Liu*

T. V. Pham, Y. Li, Dr. W. B. Luo, H. P. Guo, , Dr. X. W. Gao, Prof. J. Z. Wang, Prof. H. K. Liu

Institute for Superconducting and Electronic Materials
University of Wollongong, Wollongong, NSW 2522, Australia.
E-mail: luow@uow.edu.au; jiazhao@uow.edu.au

Key words: Ni_3Pt alloy, pulsed laser deposition, electrocatalyst, binder free, rechargeable zinc-air battery

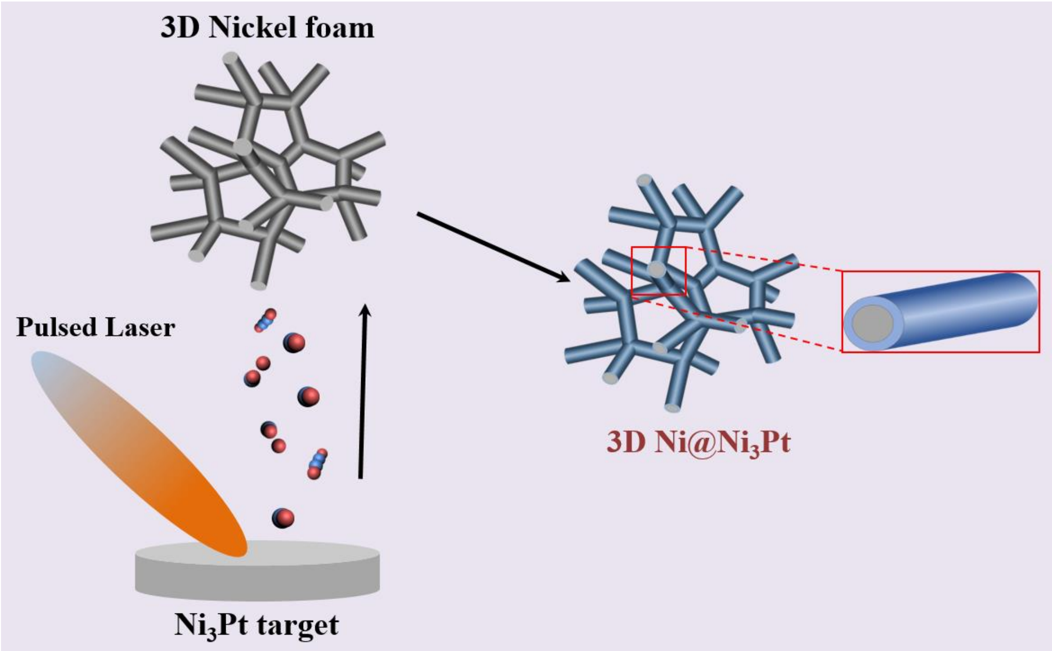

Figure S1. Schematic diagram of the experiment process.

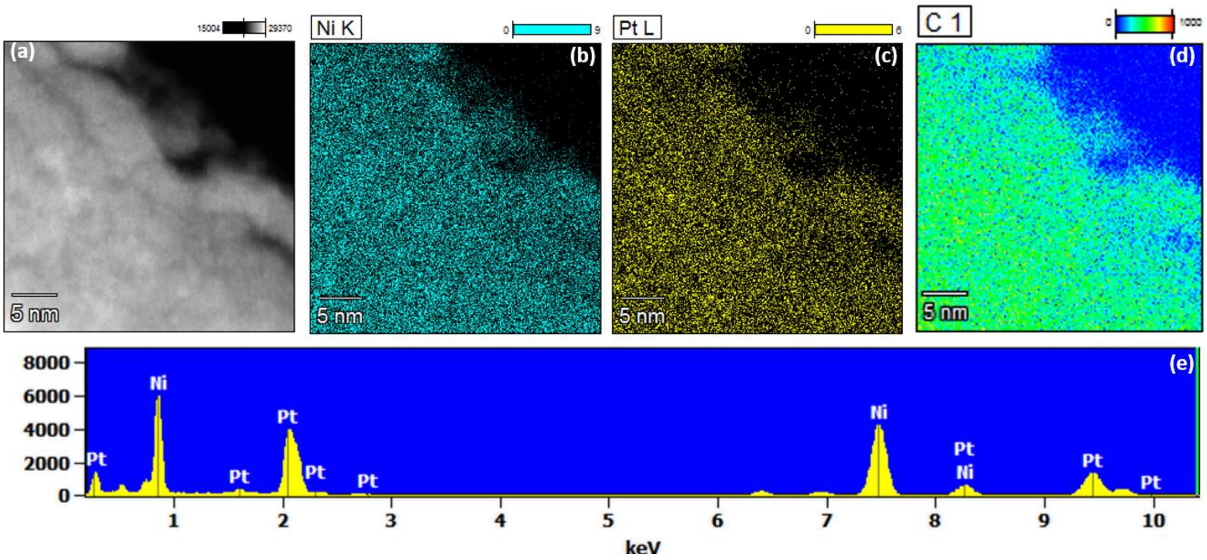

Figure S2. HAADF-STEM energy-dispersive X-ray spectroscopy element mapping of deposited Ni_3Pt thin film.

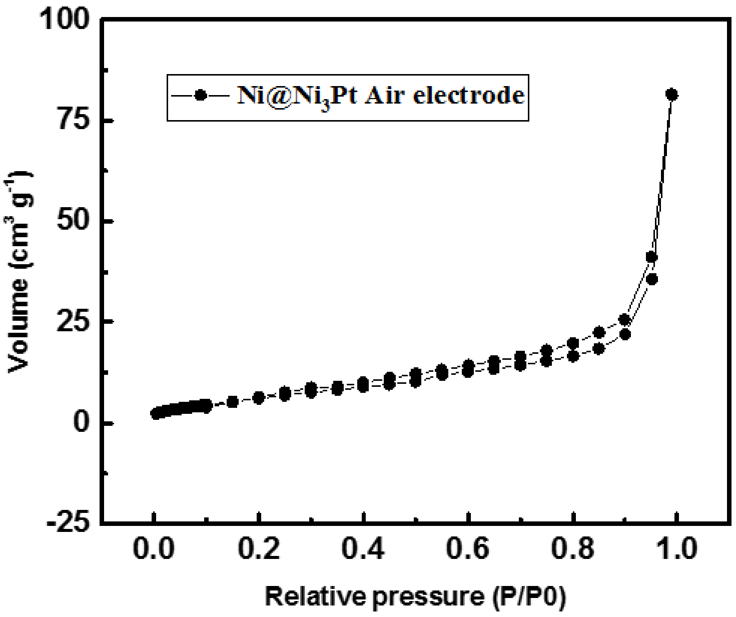

Figure S3. Brunauer–Emmett–Teller (BET) of Ni@Ni_3Pt air electrode.

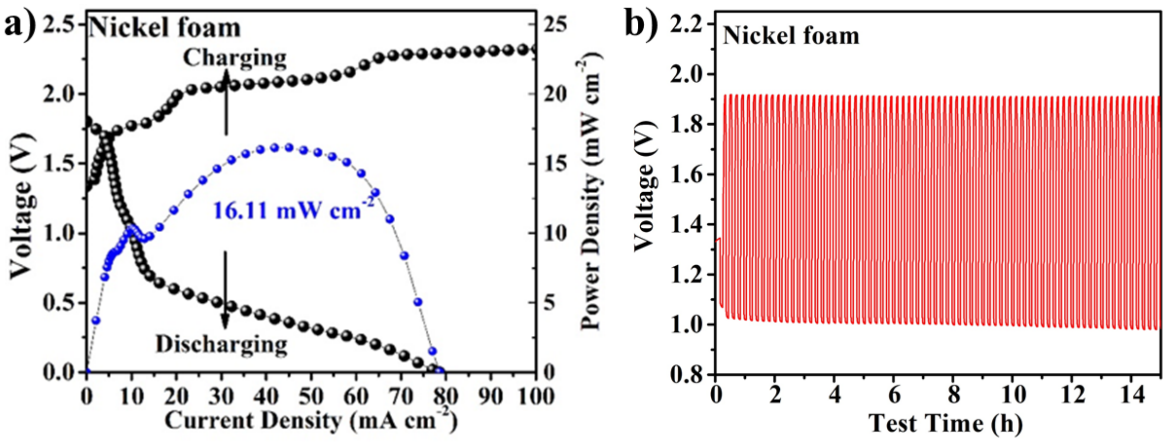

Figure S4. The electrochemical performance of pure nickel form (a) Polarization and power density curves. (b) Cycling performance at a charge-discharge current density of 10 mA cm^{-2}.

Table S1. Performance of this work secondary zinc air battery compared with other noble metal catalysts and other significant electrocatalysts.

Catalyst	Electrolyte	Charge Potential (V)	Charge/discharge overpoential (V)	Cycling Condition (mA cm^{-2})/(mA g^{-1})	Stability	Reference
Ni_3Pt on Ni Foam	6 M KOH+0.2 M $Zn(Ac)_2$	1.7	0.62	10	10 min/cycle for 478 cycles (up to 79 hours). overpotenial increased to 0.62 V	This work
Ag-Cu on Ni foam	6 M KOH+0.2 M $ZnCl_2$	2.05	0.96	20	20 min/cycle for 100 cycles with negligible voltage change	[1]
Pd_3Pb	6 M KOH+0.2 M $ZnCl_2$	2.1	072	10	240 min/cycle for 137 cycles, overpotential increased to 0.86 V	[2]
RuO_2-MCNA	6 M KOH	2	0.8	4	120 min/cycle for 80 cycles, with negligible voltage change	[3]
Ag-Cu nanoalloys on Ni foam	6 M KOH+0.1 M $Zn(Ac)_2$	2.2	1.12	20	30 min/cycle for 252 cycles with negligible voltage change	[4]
Ag decorated $LaMnO_3$ nanorod/graphene	6 M KOH	2	0.86	25	60min/cycle for 80 cycles, overpotential increased to 0.95V	[5]
Atomically coupled Pt Nanoparticles(NPs) and single-crystal CoO nanorod	3 M KOH+0.2 M Zn(Ac)2	2.2	1.2	5 mA cm^{-2} for discharge and 10 mA cm^{-2} for charge	30 cycles with negligible voltage change	[6]
NGM-Co	6 M KOH+0.2 M $ZnCl_2$	2	1	2	20 min/cycle for 180 cycles, overpotential increased to 1.12 V	[7]
CoO/N-CNT+NiFe LDH	6 M KOH+0.2 M $Zn(Ac)_2$	2	0.7	20	10 cycles with negligible voltage change	[8]
N and P co-doped porous carbon	6 M KOH	2.25	0.95	2	10 min/cycle for 600 cycles	[9]
$NiCo_2O_4$/NiF@C	6 M KOH+0.2 M $Zn(Ac)_2$	2	1	5	30 min/cycle for >5000 cycles	[10]
CuCo2O4@C	6M KOH	1.9	0.79	10	30 min/cycle for >5000 cycles	[11]
PC and QAFC membranes	1 M KOH in nanoporous cellulose membrane	2	0.8	250 mA g^{-1}	60 min/cycle for 1000 mins	[12]

Reference

[1] Y. Jin, F. Chen, *Electrochimica Acta* **2015**, 158, 437.
[2] Z. Cui, H. Chen, M. Zhao, F. J. DiSalvo, *Nano Letters* **2016**, 16, 2560.
[3] Z. Guo, C. Li, W. Li, H. Guo, X. Su, P. He, Y. Wang, Y. Xia, *Journal of Materials Chemistry A* **2016**, 4, 6282.
[4] X. Wu, F. Chen, Y. Jin, N. Zhang, R. L. Johnston, *ACS Applied Materials & Interfaces* **2015**, 7, 17782.
[5] J. Hu, Q. Liu, L. Shi, Z. Shi, H. Huang, *Applied Surface Science* **2017**, 402, 61.
[6] M. Chao, L. Tao, M. Tian-Yi, W. Hui, H. Zhenpeng, Z. Yue, M. Jing, D. Xi-Wen, J. Mietek, Q. Shi-Zhang, *Advanced Materials* **2017**, 29, 1604607.
[7] T. Cheng, W. Bin, W. Hao-Fan, Z. Qiang, *Advanced Materials* **2017**, 29, 1703185.
[8] Y. Li, M. Gong, Y. Liang, J. Feng, J.-E. Kim, H. Wang, G. Hong, B. Zhang, H. Dai, *Nature Communications* **2013**, 4, 1805.
[9] J. Zhang, Z. Zhao, Z. Xia, L. Dai, *Nature Nanotechnology* **2015**, 10, 444.
[10] B. Li, J. Quan, A. Loh, J. Chai, Y. Chen, C. Tan, X. Ge, T. S. A. Hor, Z. Liu, H. Zhang, Y. Zong, *Nano Letters* **2017**, 17, 156.
[11] X. Wang, Y. Li, T. Jin, J. Meng, L. Jiao, M. Zhu, J. Chen, *Nano Letters* **2017**, 17, 7989.
[12] J. Fu, J. Zhang, X. Song, H. Zarrin, X. Tian, J. Qiao, L. Rasen, K. Li, Z. Chen, *Energy & Environmental Science* **2016**, 9, 663.
